# Granger Causality and Jensen–Shannon Divergence to Determine Dominant Atrial Area in Atrial Fibrillation

**DOI:** 10.3390/e20010057

**Published:** 2018-01-12

**Authors:** Raquel Cervigón, Francisco Castells, José Manuel Gómez-Pulido, Julián Pérez-Villacastín, Javier Moreno

**Affiliations:** 1 Escuela Politécnica, Universidad de Castilla-La Mancha, Camino del Pozuelo sn, 16071 Cuenca, Spain; 2 Instituto ITACA, Universitad Politécnica de Valencia, 46022 Valencia, Spain; 3 Escuela Politécnica Superior, Universidad De Alcalá, Ctra. Madrid-Barcelona, Km. 33,600, 28871 Alcalá de Henares, Madrid, Spain; 4 Unidad de Arritmias, Hospital Clínico San Carlos, Plaza de Cristo Rey sn, 28040 Madrid, Spain; 5 Unidad de Arritmias, Hospital Ramón y Cajal, Ctra. de Colmenar Viejo km. 9,100, 28034 Madrid, Spain

**Keywords:** atrial fibrillation, ablation, causality, divergence

## Abstract

Atrial fibrillation (AF) is already the most commonly occurring arrhythmia. Catheter pulmonary vein ablation has emerged as a treatment that is able to make the arrhythmia disappear; nevertheless, recurrence to arrhythmia is very frequent. In this study, it is proposed to perform an analysis of the electrical signals recorded from bipolar catheters at three locations, pulmonary veins and the right and left atria, before to and during the ablation procedure. Principal Component Analysis (PCA) was applied to reduce data dimension and Granger causality and divergence techniques were applied to analyse connectivity along the atria, in three main regions: pulmonary veins, left atrium (LA) and right atrium (RA). The results showed that, before the procedure, patients with recurrence in the arrhythmia had greater connectivity between atrial areas. Moreover, during the ablation procedure, in patients with recurrence in the arrhythmial both atria were more connected than in patients that maintained sinus rhythms. These results can be helpful for procedures designing to end AF.

## 1. Introduction

Atrial fibrillation (AF) is already the most commonly occurring arrhythmia and its prevalence affects approximately 3% in adults aged 20 years or older [1], increasing the prevalence in older people. Moreover, AF set to increase owing to widespread population ageing [2,3]. Additionally, cardiovascular morbidity attributed to AF warrants particular attention. This arrhythmia is one of the main causes of stroke, embolism, sudden death or heart failure [4].

Despite AF epidemic nature and the large number of studies performed over the last decades, the mechanisms underlying AF initiation and maintenance are still not completely understood [5].

Although AF has highly variable activation, evidence exists that indicate that AF exhibits underlying spatial–temporal organization, including consistent activation vectors [6] and evidence for stable rotors and focal drivers on specific areas of the myocardium responsible for the self-perpetuating nature of AF [7,8].

During AF, pulmonary vein (PV) ostia may facilitate the re-entry process by providing areas of conduction block [9,10]. The ablation of ectopic foci in the PVs that trigger AF in some patients may provide long-term correction of AF [9].

Rhythm control can be achieved with either antiarrhythmic drug therapy or nonpharmacologic methods, including catheter-based radiofrequency ablation. This therapy has transformed from an extraordinary procedure into a frequent treatment option for patients with paroxysmal or persistent AF [11], with higher efficiency in maintaining sinus rhythm and a similar complication rate for antiarrhythmic drugs [12,13]. However, this procedure is effective in only a subset of patients and sinus rhythm maintenance rises from about 50% to just under 70% of patients with persistent AF and paroxysmal AF, respectively, without severe symptomatic recurrences of AF [14,15]. Nevertheless, no consensus has been attained on which areas should be ablated and the success rate of a single procedure is not satisfactory yet.

Previous studies have identified multiple risk factors for AF recurrence after a successful catheter ablation, including the type of AF (paroxysmal, persistent, or chronic), the duration of chronic AF, the presence of concomitant valvular heart disease and the size of the left atrium; nevertheless, their predictive power is weak [16]. Unfortunately, the rapid and seemingly chaotic electrogram activity that is characteristic of AF can not currently be used to determine whether AF in a particular patient has a PV origin or is maintained by other foci/mechanisms [17]. Nevertheless, other studies have applied Full Conditional Granger causality to find the hierarchical activation patterns between neighbouring electrodes’ signals to identify predominant propagation directions and atrial sources during AF, and more recent studies have achieved identifying dominant propagation patterns during electrical pacing by causality analysis, where these sources were consistent with location of the stimulating catheter [18,19]. However, other studies proposed a mathematical model based on Hierarchical Granger Causality analysis, where resulting causality maps contain important information about the propagation of wavefronts as well as the directions and causal connections [20,21].

The algorithm initially selects the node with the highest number of potential causal links as the root node.

Moreover, other studies evaluate directional coupling between multiple signals in the frequency domain by partial directed coherence function. It reflects the direction of coupling and thus the propagation between recording sites [22,23]. Furthermore, a recent work introduces Granger causality to analyze atrial activity interactions between different intracardiac sites during AF, providing information about atrial rhythm complexity [24].

Granger Causality is proposed as an algorithm for determining whether one time series recorded at the atrial area is useful in forecasting another from a different area. In addition, Jensen–Shannon divergence was proposed as a novel method to analyze distance between signals. This method has already been applied to a variety of different disciplines, from information theory and mathematical statistics [25] to biomedical engineering. A very recent study applied this divergence to explore the relationship between eye movements and electrocardiogram interpretation accuracy, providing information that can help to improve both human and automated interpretation approaches [26]. Other studies apply this method to simulated and real life signals, showing in all cases a high proficiency for detecting changes in the dynamics of the associated time series [27,28].

In this study, we analysed atrial electrical activity collected before and during PV isolation procedure. Data on the recurrence after treatment during the first year after the isolation procedure divided patients into two different groups, sinus rhythm maintained patients and recurrent AF patients. We studied causal interdependencies and couplings between different atrial regions to uncover new insights into the dynamics of AF, with the objective of analysing if there are differences between recurrent and non-recurrent AF patients.

The aim of this paper is to prove that causality and divergence theories application can accurately capture spatial and temporal cardiovascular signals fluctuations from atrial structures to provide information that can be used to improve the optimal approach to catheter ablation.

## 2. Materials

Ten patients with AF were submitted to a catheter ablation procedure and intracardiac recordings were obtained immediately before and during the intervention. All patients were monitored after the procedure and divided into two groups according to long-term ablation success, defined as the absence of arrhythmia 12 months after the ablation. In addition, several parameters are showed in Table 1 such as age, sex, left atrium size, structural cardio-pathology (SC), arterial hypertension (AH), electrical cardioversion (ECV), AF duration longer than six months and patients with paroxysmal AF, using either one (1) or zero (0), expressing “true” or “false”, respectively.

**Figure 1:**
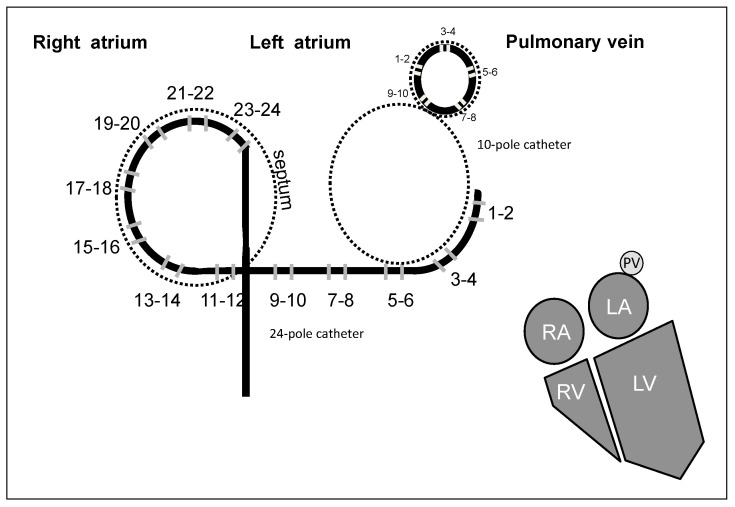
Diagrammatic representation illustrating Orbiter and Lasso catheters’ distribution of electric poles along both atria and a pulmonary vein. The lower right figure represents the anatomic relation of the four cardiac heart chambers, showing atria and ventricles and one pulmonary vein.

A 12-bipolar catheter (Orbiter PV, Bard Electrophysiology, Lowell, MA, USA; 2–9–2 mm electrode spacing) was inserted through the femoral vein and positioned in the right atrium (RA) with the medium and proximal group of electrodes located spanning the RA free-wall peritricuspid area, from the coronary sinus ostium to the upper part of the interatrial region left atrial (LA) electrical activity as well (Figure 1). Twelve bipolar intracardiac electrograms were digitally recorded at 1 kHz sampling rate (16 bit A/D conversion; Polygraph Prucka Cardio-Lab, General Electric, Piscataway, NJ, USA) from the RA (dipoles from 14–15 to 23–24) and the LA (dipoles 1–2, 3–4, 5–6 and 7–8) using this catheter.

Additionally, a 10-pole catheter (Lasso, 2-5-2 mm electrode spacing, Biosense Webster, Irwindale, CA, USA) pulmonary vein sized loop-shaped, was introduced via a transatrial septal long sheath, five bipolar intracardiac electrograms from each PV were digitally recorded at 1 kHz sampling rate (16 bit A/D conversion; Polygraph Prucka Cardio-Lab, General Electric, Piscataway, NJ, USA) (Figure 1).

Fifty to sixty second Lasso and Orbiter catheters recordings from paroxysmal and persistent AF patients were recorded before the ablation. The orbiter catheter was at the same location during the entire procedure; nevertheless, the Lasso catheter was changing from the right superior pulmonary vein (RSPV), the right inferior pulmonary vein (RIPV), the left inferior pulmonary vein (LIPV), to the left superior pulmonary vein (LSPV) and, as a result, we had recordings from the four PVs before the procedure. Moreover, fifty to sixty second Orbiter catheter recordings were recorded along different phases during the intervention: in the basal state, after isolation of right PVs, after left PVs’ isolation and at the end of the procedure.

## 3. Methods

In this section, we present the methods applied to time series analysis. The major challenge to capture the temporal dependence between different areas in recurrent AF patients and patients that maintain sinus rhythm. We propose a simple yet extremely effective approach by extracting the main component with Principal Component Analysis (PCA) and study causal and divergence relations between different areas, before and during ablation procedure.

### 3.1. Principal Component Analysis

PCA is applied in order to remove the redundancy of the electrogram. It extracts the most important variables, in the form of principal components (PCs) from a large set of signals available in the electrogram. PCA is a popular data processing and dimension reduction technique [29]. The first PC is the linear combination of the entrance variables that has maximum variance (among all linear combinations), so it accounts for as much variation in the data as possible.

**Figure 2:**
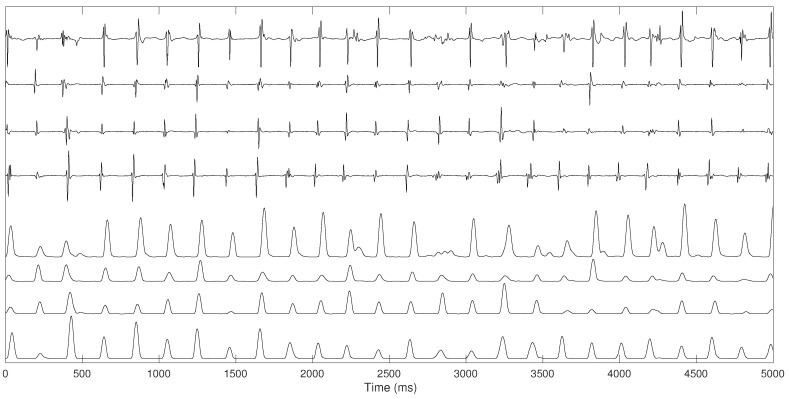
Four original RA electrogram signals (**top**) and pre-processing signals using the Botteron pre-processing chain (**bottom**).

PCA was applied for the signals recorded with Orbiter and Lasso catheters before and during the ablation procedure and the first component was extracted. The analysis was done in two phases:

- Bipolar electrogram signals were pre-processed using several steps proposed by Botteron [30]. Initially, the signals were band-pass filtered between 40 and 250 Hz. Subsequently, they were rectified and, finally, the absolute value of the filtered waveforms were lowpass filtered with a 20-Hz cut-off filter. This filtering process extracts a time-varying waveform proportional to the amplitude of the high-frequency components of the original atrial electrograms, enhancing the atrial activations, simplifying their shape variations while reducing noise, as illustrated in Figure 2.
- PCA was applied to the pre-processed recordings on the RA and on the LA with Orbiter catheter and on the PVs with Lasso catheter before ablation procedure. We extract the first PC from the four electrograms recorded on the four PVs, simultaneous to the recordings from the LA and the RA, where the first PC was extracted in each area.
- PCA was applied to the recordings recorded on the LA and on the RA with the Orbiter catheter in four phases: in basal state, after right PVs’ isolation, after left PVs’ isolation and after the ablation procedure.

Only the first PC was analyzed since it contained more than 50% of the total variance and it was related to the main information, whereas the last components were associated with noise and redundancy.

#### Suitability of PCA Decomposition

Representative LA and RA activation sequences are obtained after PCA decomposition from the EGMs at each atrium. As the atrial activations are slightly delayed from one site to another, the suitability of PCA decomposition is studied in this section. Let us consider several signals with randomly delayed pulses. The shorter the delays, the higher the overlap of the pulses and, hence, the higher the correlation between the signals. Accordingly, the first component of the PC decomposition will exhibit a higher variance content.

**Figure 3:**
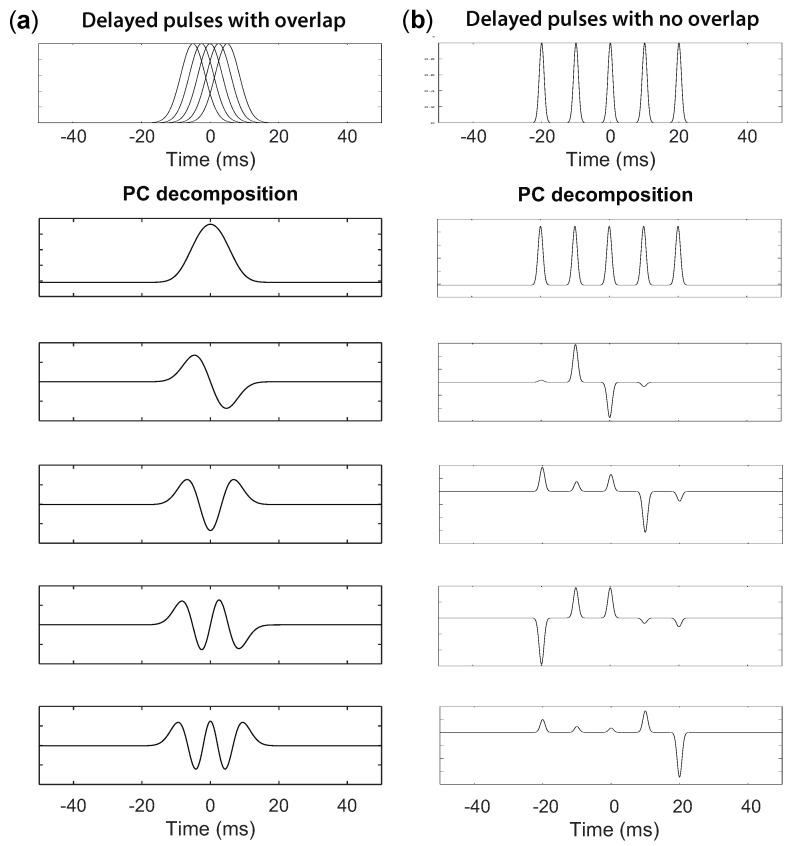
Five Gaussian-shaped pulses with increasing delays (**a**) and PC decomposition (**b**).

On the other hand, when there is no overlap between the activation pulses, the PCA decomposition is no longer valid. As shown in Figure 3b, the first PC is the average of the signals and becomes a sequence of pulses. The following PCs exhibit different weight combinations at each pulse, with the restriction that all weights’ vectors are orthogonal among them. As a result, no interesting information can be retrieved from these transformations.

Figure 3a shows five Gaussian-shaped pulses with increasing delays (this shape resembles the pulse shape of the EGM pulses after being pre-processed according to the steps proposed by Botteron [31]). PC decomposition is shown, when the delay between the first and the last pulses is half the pulse duration. On the other hand, when there is no overlap between the activation pulses, the PCA decomposition is no longer valid. As shown in Figure 3b, the first PC is the average of the signals and becomes a sequence of pulses. The following PCs exhibit different weight combinations at each pulse, with the restriction that all weights’ vectors are orthogonal among them. As a result, no interesting information can be retrieved from these transformations.

The first component is the mean of the signals, with a variance content of 69.5%. The second component is the derivative of the first component, and its variance content is up to 25.3%. Subsequently, each component corresponds to the first derivative of the preceding one, with a decreasing variance content.

**Figure 4:**
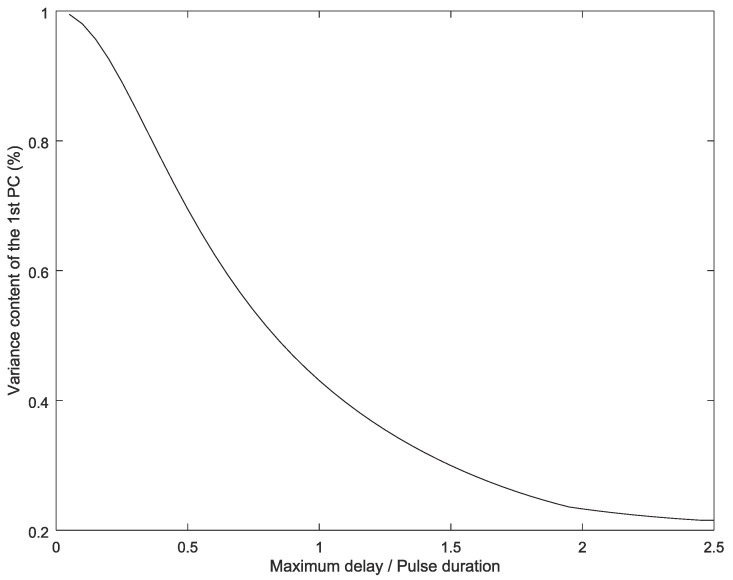
Variance content of the first component vs. delay between activations.

As the delays between activations increases, the variance content of the first component decreases progressively (Figure 4). Asymptotically, the variance content tends to a fifth, as all signals become almost uncorrelated, and, therefore, all principal components present the same variance content.

### 3.2. Granger Causality

Granger causality (G-Causality) analysis has been one of the most broadly-established instruments of identifying causal relations between two times series. It has become increasingly used in many knowledge areas including biomedical signal processing [32,33,34]. G-Causality is defined in terms of upgrading predictability and as a measure of directed functional connectivity. This method was applied to the first PC extracted with PCA. The implementation of G-Causality is via vector autoregressive modeling, in which PCs’ time series from different regions are modelled as weighted sums of the past values.

Given two time series **X** = $X_{i}$: *i*≥ 1 and **Y** = $Y_{i}$: *i*≥ 1, to determine whether **X** causes **Y**, **Y** is first modelled as an univariate autoregressive series with error correction term $V_{i}$:

(1) $$Y_{i}=\sum_{j=1}^{p}a_{j}Y_{i-j}+V_{i}.$$

Then, **Y** is modelled again, using the **X** series as causal side information:

(2) $$Y_{i}=\sum_{j=1}^{p}\left[b_{j}Y_{i-j}+c_{j}X_{i-j}\right]+\tilde{V_{i}},$$

with $\tilde{V_{i}}$ as the new error correction term. The value of *p* was fixed a priori or determined using Akaike information criterion order selection tool [35]. The G-Causality is defined as:

(3) $$G_{X\to Y}\equiv log\frac{var(V)}{var(\tilde{V}).}$$

This technique evaluates the ratio of the variances of the correction terms. $G_{X\to Y}$ and $G_{Y\to X}$ are compared, where the larger term is taken to be the direction of causal influence.

### 3.3. Jensen–Shannon Divergence

Several measures have been proposed to quantify the difference sometimes called divergence between two or more probability distributions [36]. One of those measures is the Jensen–Shannon divergence (JSD), defined as a distance measure between probability distributions introduced by Rao [37] and Lin [38] as a symmetric version of the Kullback–Leibler divergence. This measure diverges when the two distributions are disjoint.

The Shannon entropy (H(P)) for the probability distribution *P* defined by Equation (4), where $P_{i}$ are probability distributions:

(4) $$H\left(P\right)=-\sum_{i=1}^{N}p_{i}log\left(p_{i}\right).$$

All distances in this section are defined on the set **Z** of all variables, where X,Y ∈ **Z**. Evaluating the proximity of probability distributions $P_{1}$ and $P_{2}$, with $X_{i}$, i=1,2,⋯,N and $Y_{i}$, i=1,2,⋯,N, which we denote as $p_{i}^{\left(1\right)}=P_{1}\left(X_{i}\right)$ and $p_{i}^{\left(2\right)}=P_{2}\left(Y_{i}\right)$, with $0\leq p_{i}^{\left(k\right)}\leq 1$ and $\sum_{i=1}^{N}p_{i}^{\left(k\right)}=1$ for all i=1,2,⋯,n and k=1,2. If $\pi_{1}$ denotes the weight of $P_{1}$ and $\pi_{2}$ is the weight $P_{2}$, with the restrictions $\pi_{1}+\pi_{2}=1$ and $\pi_{1},\pi_{2}\geq 0$, the JSD is defined by:

(5) $$D_{JS}\left(P_{1},P_{2}\right)=H\left(\pi_{1}P_{1}+\pi_{2}P_{2}\right)-\left(\pi_{1}H\left(P_{1}\right)+\pi_{2}H\left(P_{2}\right)\right).$$

It is possible to observe that the JSD is positive defined, symmetric and it is zero if and only if $P_{1}=P_{2}$ [39]. Moreover JSD is the square of a metric [40] and it can be generalized in order to compare an arbitrary number of distributions, in the following way: let $P_{1}\left(x\right),\cdots ,P_{M}\left(x\right)$ be a set of probability distributions with j=1,⋯,M and let $\pi_{1}\cdots \pi_{M}$ be a set of non negative weights such that $\sum_{j=1}^{M}\pi_{j}=1$. Then, the JSD is defined by:

(6) $$D_{JS}\left(P_{1},\cdots ,P_{M}\right)=H\left[\sum_{j=1}^{M}\pi_{j}P_{j}\right]-\sum_{j=1}^{M}\pi_{j}H\left[P_{j}\right].$$

### 3.4. Statistical Analysis

All data are expressed as median ± interquartile range (IQR). The Mann–Whitney U-test was performed to determine whether there was a significant difference between the two groups. The *t*-statistic was considered not significant at the 0.05 critical alpha level, p<0.05.

## 4. Results

Electrograms recorded before the ablation procedure and during the ablation procedure were analysed. In the first case, we analysed Lasso catheter recordings from PVs and Orbiter catheter recordings from the LA and the RA. In the second study, we had orbiter recordings from both atria obtained along the ablation procedure.

In the first study, PCA was applied and the first component from each PV and from the RA and the LA were extracted, and, in the second study, the first PC from the LA and the RA were extracted and analysed.

### 4.1. Analysis Pre-Ablation Procedure

We analysed the first PC from each PV, and, at the same time, the 1st PC from the RA and the 1st PC from the LA that were recorded simultaneously.

#### 4.1.1. Granger Causality

**Figure 5:**
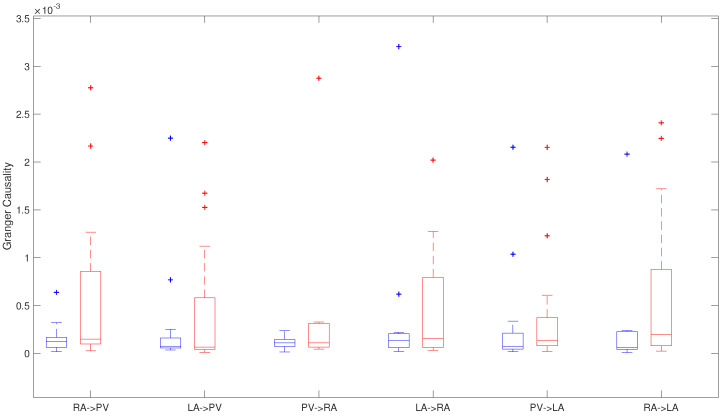
G-Causality in relation to atrial area in AF recurrent patients (red) and patients that maintain sinus rhythm (blue).

Inter-regional atrial activity relation has been studied before ablation procedure using G-Causality analysis. If the first PC from a region, **X**, is causally driving **Y** (first PC from another region), in the recurrent and non-recurrent AF patients (Figure 5).

This study showed differences between both groups along different regions. Nevertheless, only interaction between both chambers showed statistical significant differences between both groups, with higher data dispersion in the patients that had recurrence in AF (Figure 5). RA → LA G-causality with $0.62\times 10^{-4}$ ± $1.87\times 10^{-4}$ in patients maintaining normal sinus rhythm and $1.97\times 10^{-4}$ ± $7.97\times 10^{-4}$ in patients with recurrent AF, respectively (p=0.047). Moreover, RA → PV G-causality was from $1.21\times 10^{-4}$ ± $1.04\times 10^{-4}$ in the non-recurrent AF group to $1.50\times 10^{-4}$ ± $7.58\times 10^{-4}$, in the recurrent AF patients (p=0.099).

Moreover, it is possible to observe differences on G-causality along different areas in recurrent AF patients and in patients in whom normal rhythm is maintained, where the main differences are found between RA → PV and RA → LA (Table 2).

#### 4.1.2. Jensen–Shannon Divergence

**Figure 6:**
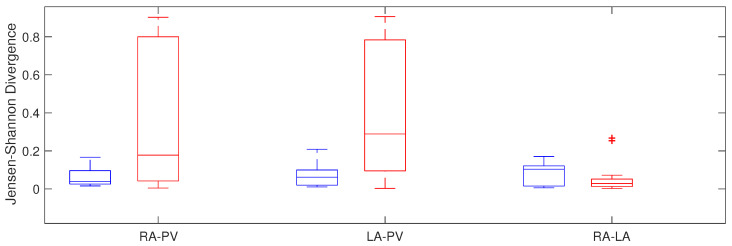
Jensen–Shannon divergence in relation to atrial area in recurrent AF patients (red) and patients that maintain sinus rhythm (blue).

Jensen–Shannon Divergence showed differences between RA and PV, with 0.04 ± 0.07 in the non recurrent AF patients and 0.18 ± 0.76 in the recurrent AF patients (*p* = 0.015) and between left chamber and pulmonary veins, with 0.06 ± 0.08 vs. 0.29 ± 0.69 in patients with recurrent AF and in patients maintaining sinus rhythm, respectively (p< 0.001), with higher dispersion in the group of patients that did not maintain normal rhythm. Moreover, non statistical significant differences were obtained between both atria (Figure 6).

### 4.2. Analysis during the Ablation Procedure

During the ablation procedure the first PC from the LA and from the RA were extracted in four different phases:

- Phase 1: Basal state;
- Phase 2: After right PVs’ isolation;
- Phase 3: After left PVs’ isolation;
- Phase 4: After the procedure.

G-causality and divergence were studied across the atria during the different phases of the ablation procedure.

#### 4.2.1. Granger Causality

**Figure 7:**
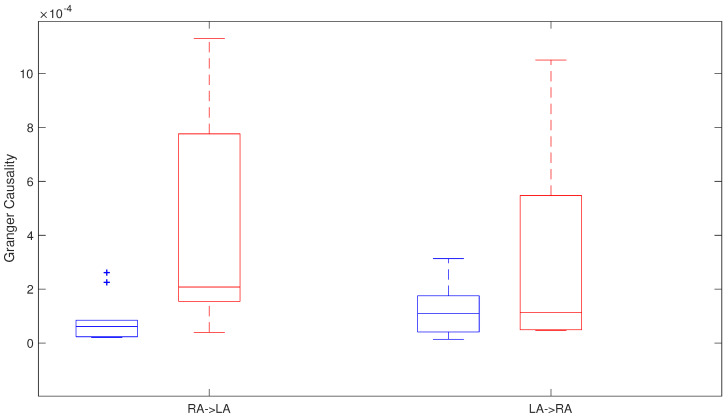
Granger causality in relation to all the phases in recurrent AF patients (red) and patients that maintain sinus rhythm (blue).

Granger causality was applied along the four phases and global differences from RA → LA were found between both groups (p= 0.031) with G-causality values from $0.61\times 10^{-4}$ ± $0.61\times 10^{-4}$ to $2.08\times 10^{-4}$ ± $6.22\times 10^{-4}$, in the group maintaining normal sinus rhythm and in the group with AF recurrence, respectively. Moreover, individual phases analysis showed that, during the first phase and the last phase, there were G-causality differences between both groups. G-causality values during the last phase were from $0.63\times 10^{-4}$ ± $0.70\times 10^{-4}$ in patients that maintained a sinus rhythm to $1.92\times 10^{-4}$ ± $4.25\times 10^{-4}$ in recurrent AF patients (p= 0.044), with the same trend in the two previous phases, but not reaching significance. Furthermore, G-causality LA → RA differences were smaller, with not statistically significant differences between both groups in any phase, or when the analysis included data from the four phases (p= 0.371) with $1.09\times 10^{-4}$ ± $1.34\times 10^{-4}$ in the non-recurrent AF group and $1.11\times 10^{-4}$ ± $4.98\times 10^{-4}$ in the recurrent AF patients (Figure 7).

#### 4.2.2. Jensen–Shannon Divergence

**Figure 8:**
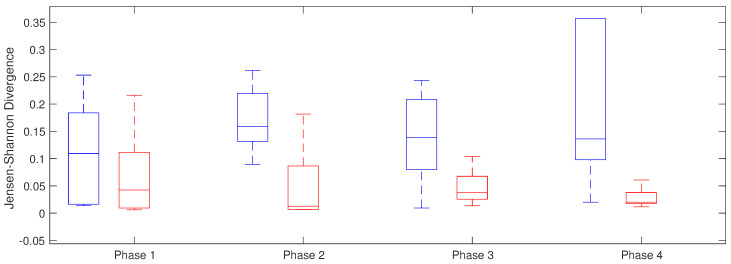
Jensen–Shannon Divergence between LA and RA along the for phases in recurrent AF patients (red) and patients in sinus rhythm (blue).

Jensen–Shannon divergence was applied to study inter-atrial divergence along all the phases in both groups. The highest difference was from the isolation of right PVs (phase 2) until the end of the intervention, with statistically significant differences between both groups. These results are showed in Table 3 with higher values in the group that maintain sinus rhythm than in the patients that have recurrence in the arrhythmia (Figure 8).

## 5. Discussion

Pulmonary vein catheter ablation is a widely used procedure that isolates the pulmonary veins from the rest of the heart and prevents any pulses from these veins from getting into the heart, restoring normal rhythm in patients with AF [41].

However, it has a limited overall success rate and frequently it is necessary to repeat the procedure. These limitations can be caused by our current poor understanding of the pathophysiology of the arrhythmia. Nevertheless, previous studies have described numerous clinical variables that were identified as independent predictors of recurrence of AF: non-paroxysmal AF, aging, left ventricular systolic and diastolic dysfunction, LA enlargement, the presence of non-PV triggers, obesity, and hypertension [42,43].

In this study, we analyse ten patients’ registers before and during AF ablation procedure. PVs’ bipolar electrograms were recorded to analyse electrical activity because its abnormal activation, with slow and anisotropic conduction, results in pro-arrhythmic activity [44] that is directly responsible for the generation of AF in many patients [9]. In addition, we recorded atrial electrical activity by a bipolar electrogram along both atria. A new approach combining PCA has been proposed to reduce dimension of the data and Granger causality and divergence to analyse connectivity between different atrial regions. Considering that, during AF, atria is a complex system that consists of many parts or subsystems that interact in a complex way. A dataset represents the temporal dynamics of possibly interacting variables recorded from possibly interacting subsystems. Our goal is to estimate whether there exists any causal relationship between two or more of these variables.

Results before ablation procedure showed higher divergence between PVs and atria in the patients with recurrence of AF than in patients whose rhythm is regular. Nevertheless, Granger causality showed differences in the causality from RA to LA, and our results showed a lower connectivity between RA and LA in patients with no AF recurrence. This could be explained by the fact that pulmonary vein isolation is carried out at the left atrium. This is in patients with no AF recurrence, and the drivers were localized at the left atrium, so that the expected causality is in the direction LA → RA than RA → LA. These results are consistent with previous literature findings, where a significantly lower prevalence of AF foci in the LA and PVs and higher prevalence of AF foci in the RA was related with recurrence [45]. Certainly, initiation and perpetuation of AF may be fairly dependent on the anatomic and functional obstacles that exist in the right chamber. Moreover, experimental studies suggest that focal drivers of AF or stable or unstable re-entry circuits may be located in different critical regions of the atria [46].

Moreover, analysis during the procedure follows the same trend observed in previous results. They showed higher connectivity between the LA and the RA and higher data dispersion in the recurrent AF patients, with higher divergence between both atria along all the phases in the non-recurrent AF patients. These results are in accordance with a previous study where entropy measurements of intracardiac signals recorded in both atria showed higher differences between both atria in patients that maintained normal rhythm and more irregular atrial electrical activity [47]. Furthermore, it is observed that the coronary sinus musculature provides a connection between the right and the left atrium as well as a part of the interatrial electrical connection [48], and in some patients with AF, rapid repetitive activity in the musculature of the coronary sinus may contribute to perpetuating the arrhythmia [49].

Understanding the pathophysiological mechanisms that reflect the interactions across atria during AF can be very helpful to locate focus areas and to know which is the best procedure to maintain the sinus rhythm after a successful ablation procedure. Using mathematical tools such as Granger causality can be helpful to understand the behaviour of the atrial interactions across different patients.

## 6. Conclusions

In conclusion, the proposed method has a major strength—the capability to perform a direct connectivity relations between different anatomical regions, such as atrial chambers and PVs. This algorithm might be used by the physician to provide some prospective measure of ablation outcome, where higher G-causality from the right to the left atrium and lower divergence between both atria could be indicators for AF recurrence after pulmonary veins’ isolation.

## Figures and Tables

**Table 1:** Patients’ characteristics.

Patient	Age	Sex	LA Size	SC	AH	AF >	ECV	Paroxysmal	Recurrence
	(Years)		(mm)			6 Months		AF	
Pat 1	67	Male	42	0	0	0	1	0	0
Pat 2	63	Female	48	0	1	0	1	1	1
Pat 3	32	Male	42	0	0	0	0	1	0
Pat 4	52	Male	45	0	1	0	0	0	0
Pat 5	65	Female	45	0	0	1	1	0	0
Pat 6	24	Male	36	0	0	0	1	1	1
Pat 7	51	Male	54	0	1	1	1	0	1
Pat 8	39	Male	35	0	0	0	1	1	0
Pat 9	57	Female	50	0	0	0	0	1	1
Pat 10	38	Male	41	0	0	1	1	0	1

**Table 2:** G-Causality along three different anatomical areas in patients that maintain sinus rhythm and in patients with recurrent AF.

G-Causality	Recurrent AF	Non Recurrent AF	*p*
**RA → VP**	$1.21\times 10^{-4}$ ± $1.04\times 10^{-4}$	$1.50\times 10^{-4}$ ± $7.58\times 10^{-4}$	0.099
**LA → VP**	$0.76\times 10^{-4}$ ± $1.04\times 10^{-4}$	$0.65\times 10^{-4}$ ± $6.45\times 10^{-4}$	0.433
**PV → RA**	$1.12\times 10^{-4}$ ± $0.75\times 10^{-4}$	$1.11\times 10^{-4}$ ± $2.57\times 10^{-4}$	0.499
**LA → RA**	$1.39\times 10^{-4}$ ± $1.51\times 10^{-4}$	$1.56\times 10^{-4}$ ± $7.34\times 10^{-4}$	0.499
**PV → LA**	$0.73\times 10^{-4}$ ± $1.78\times 10^{-4}$	$1.39\times 10^{-4}$ ± $3.25\times 10^{-4}$	0.181
**RA → LA**	$0.62\times 10^{-4}$ ± $1.87\times 10^{-4}$	$1.97\times 10^{-4}$ ± $7.97\times 10^{-4}$	0.047

**Table 3:** Jensen–Shannon Divergence along AF ablation procedure in non recurrent and recurrent AF patients.

Phases	Non Recurrent AF	Recurrent AF	*p*
	RA-LA JSD	RA-LA JSD	
Phase 1	0.11 ± 0.17	0.04 ± 0.10	0.420
Phase 2	0.16 ± 0.09	0.012 ± 0.08	0.042
Phase 3	0.14 ± 0.13	0.04 ± 0.04	0.122
Phase 4	0.14 ± 0.26	0.02 ± 0.02	0.044

## Abbreviations

The following abbreviations are used in this manuscript:

AH	Arterial Hypertension
AF	Atrial Fibrillation
CS	Coronary Sinus
ECV	Electrical Cardioversion
G-Causality	Granger Causality
JSD	Jensen–Shannon Divergence
IQR	Interquartile Range
LA	Left Atrium
LIPV	Left Inferior Pulmonar Vein
LSPV	Left Superior Pulmonar Vein
PCA	Principal Component Analysis
PC	Principal Component
PV	Pulmonar Vein
RA	Right atrium
RIPV	Right Inferior Pulmonar Vein
RSPV	Right Superior Pulmonar Vein
SC	Structural Cardiopathology

## References

[1] Haim M., Hoshen M., Reges O., Rabi Y., Balicer R., Leibowitz M. (2015). Prospective national study of the prevalence, incidence, management and outcome of a large contemporary cohort of patients with incident non-valvular atrial fibrillation. J. Am. Heart Assoc..

[2] Kirchhof P., Benussi S., Kotecha D., Ahlsson A., Atar D., Casadei B., Castellá M., Diener H.C., Heidbuchel H., Hendriks J. (2017). 2016 ESC Guidelines for the Management of Atrial Fibrillation Developed in Collaboration With EACTS. Rev. Esp. Cardiol..

[3] Chugh S.S., Havmoeller R., Narayanan K., Singh D., Rienstra M., Benjamin E.J., Gillum R.F., Kim Y.H., McAnulty J.H., Zheng Z.J. (2014). Worldwide epidemiology of atrial fibrillation: A Global Burden of Disease 2010 Study. Circulation.

[4] Fuster V., Rydén L.E., Cannom D.S., Crijns H.J., Curtis A.B., Ellenbogen K.A., Halperin J.L., Kay G.N., Le Huezey J.Y., Lowe J.E. (2011). 2011 ACCF/AHA/HRS focused updates incorporated into the ACC/AHA/ESC 2006 Guidelines for the management of patients with atrial fibrillation: A report of the American College of Cardiology Foundation/American Heart Association Task Force on Practice Guidelines developed in partnership with the European Society of Cardiology and in collaboration with the European Heart Rhythm Association and the Heart Rhythm Society. J. Am. Coll. Cardiol..

[5] Krummen D.E., Narayan S.M. (2009). Mechanisms for the initiation of human atrial fibrillation. Heart Rhythm.

[6] Gerstenfeld E.P., Sahakian A.V., Swiryn S. (1992). Evidence for transient linking of atrial excitation during atrial fibrillation in humans. Circulation.

[7] Narayan S.M., Krummen D.E., Shivkumar K., Clopton P., Rappel W.J., Miller J.M. (2012). Treatment of atrial fibrillation by the ablation of localized sources: CONFIRM (Conventional Ablation for Atrial Fibrillation With or Without Focal Impulse and Rotor Modulation) trial. J. Am. Coll. Cardiol..

[8] Shivkumar K., Ellenbogen K.A., Hummel J.D., Miller J.M., Steinberg J.S. (2012). Acute termination of human atrial fibrillation by identification and catheter ablation of localized rotors and sources: First multicenter experience of focal impulse and rotor modulation (FIRM) ablation. J. Cardiovasc. Electrophysiol..

[9] Haïssaguerre M., Jaïs P., Shah D.C., Takahashi A., Hocini M., Quiniou G., Garrigue S., Le Mouroux A., Le Métayer P., Clémenty J. (1998). Spontaneous initiation of atrial fibrillation by ectopic beats originating in the pulmonary veins. N. Engl. J. Med..

[10] Jalife J., Berenfeld O., Mansour M. (2002). Mother rotors and fibrillatory conduction: A mechanism of atrial fibrillation. Cardiovasc. Res..

[11] Arbelo E., Brugada J., Hindricks G., Maggioni A.P., Tavazzi L., Vardas P., Laroche C., Anselme F., Inama G., Jais P. (2014). The atrial fibrillation ablation pilot study: a European Survey on Methodology and results of catheter ablation for atrial fibrillation conducted by the European Heart Rhythm Association. Eur. Heart J..

[12] Walfridsson H., Walfridsson U., Nielsen J.C., Johannessen A., Raatikainen P., Janzon M., Levin L.A., Aronsson M., Hindricks G., Kongstad O. (2015). Radiofrequency ablation as initial therapy in paroxysmal atrial fibrillation: Results on health-related quality of life and symptom burden. The MANTRA-PAF trial. Europace.

[13] Mont L., Bisbal F., Hernandez-Madrid A., Perez-Castellano N., Vinolas X., Arenal A., Arribas F., Fernandez-Lozano I., Bodegas A., Cobos A. (2014). Catheter ablation vs. antiarrhythmic drug treatment of persistent atrial fibrillation: A multicentre, randomized, controlled trial (SARA study). Eur. Heart J..

[14] Ganesan A.N., Shipp N.J., Brooks A.G., Kuklik P., Lau D.H., Lim H.S., Sullivan T., Roberts-Thomson K.C., Sanders P. (2013). Long-term outcomes of catheter ablation of atrial fibrillation: A systematic review and meta-analysis. J. Am. Heart Assoc..

[15] Verma A., Jiang C.Y., Betts T., Chen J., Deisenhofer I., Mantovan R., Macle L., Morillo C., Haverkamp W., Weerasooriya R. (2015). Approaches to Catheter Ablation for Persistent Atrial Fibrillation. N. Engl. J. Med..

[16] Seaburg L., Hess E.P., Coylewright M., Ting H.H., McLeod C.J., Montori V.M. (2014). Shared decision making in atrial fibrillation: Where we are and where we should be going. Circulation.

[17] Lazar S., Dixit S., Marchlinski F.E., Callans D.J., Gerstenfeld E.P. (2004). Presence of left-to-right atrial frequency gradient in paroxysmal but not persistent atrial fibrillation in humans. Circulation.

[18] Rodrigo M., Guillem M.S., Liberos A., Millet J., Berenfeld O., Climent A.M. Identification of fibrillatory sources by measuring causal relationships. Proceedings of the 2012 Computing in Cardiology.

[19] Rodrigo M., Climent A.M., Liberos A., Calvo D., Fernández-Avilés F., Berenfeld O., Atienza F., Guillem M.S. (2016). Identification of Dominant Excitation Patterns and Sources of Atrial Fibrillation by Causality Analysis. Ann. Biomed. Eng..

[20] Luengo D., Munoz G.R., Elvira V. Causality analysis of atrial fibrillation electrograms. Proceedings of the 2015 Computing in Cardiology Conference (CinC).

[21] Luengo D., Rios-Munoz G., Elvira V., Artés-Rodríguez A. A hierarchical algorithm for causality discovery among atrial fibrillation electrograms. Proceedings of the 2016 IEEE International Conference on Acoustics, Speech and Signal Processing (ICASSP).

[22] Richter U., Faes L., Cristoforetti A., Masè M., Ravelli F., Stridh M., Sörnmo L. (2011). A novel approach to propagation pattern analysis in intracardiac atrial fibrillation signals. Ann. Biomed. Eng..

[23] Richter U., Faes L., Ravelli F., Sörnmo L. (2012). Propagation pattern analysis during atrial fibrillation based on sparse modeling. IEEE Trans. Biomed. Eng..

[24] Alcaine A., Mase M., Cristoforetti A., Ravelli F., Nollo G., Laguna P., Martinez J.P., Faes L. (2017). A Multi-Variate Predictability Framework to Assess Invasive Cardiac Activity and Interactions During Atrial Fibrillation. IEEE Trans. Biomed. Eng..

[25] Ré M.A., Azad R.K. (2014). Generalization of entropy based divergence measures for symbolic sequence analysis. PLoS ONE.

[26] Davies A., Brown G., Vigo M., Harper S., Horseman L., Splendiani B., Hill E., Jay C. (2016). Exploring the Relationship Between Eye Movements and Electrocardiogram Interpretation Accuracy. Sci. Rep..

[27] Mateos D.M., Riveaud L.E., Lamberti P.W. (2017). Detecting dynamical changes in time series by using the Jensen Shannon divergence. Chaos.

[28] Wang P., Wang J. (2014). Complexity analysis of gait signal based on Jensen-Shannon divergence. J. Biomed. Eng..

[29] Jolliffe I. (2002). Principal Component Analysis.

[30] Botteron G.W., Smith J.M. (1995). A technique for measurement of the extent of spatial organization of atrial activation during atrial fibrillation in the intact human heart. IEEE Trans. Biomed. Eng..

[31] Castells F., Cervigón R., Millet J. (2014). On the preprocessing of atrial electrograms in atrial fibrillation: understanding Botteron’s approach. Pacing Clin. Electrophysiol..

[32] Granger C. (1969). Investigating Causal Relations by Econometric Models and Cross-spectral Methods. Econometrica.

[33] Gao L., Smielewski P., Czosnyka M., Ercole A. (2017). Early Asymmetric Cardio-Cerebral Causality and Outcome after Severe Traumatic Brain Injury. J. Neurotrauma.

[34] Quinn C., Coleman T., Kiyavash N., Hatsopoulos N. (2011). Estimating the directed information to infer causal relationships in ensemble neural spike train recordings. J. Comput. Neurosci..

[35] Akaike H. (1978). A Bayesian analysis of the minimum AIC procedure. Ann. Inst. Stat. Math..

[36] Katatbeh Q., Martínez-Aroza J., Gómez-Lopera J., Blanco-Navarro D. (2015). An Optimal Segmentation Method Using Jensen–Shannon Divergence via a Multi-Size Sliding Window Technique. Entropy.

[37] Rao C. (1987). Differential Metrics in Probability Spaces. Differential Geometry in Statistical Inference.

[38] Lin J. (1991). Divergence measures based on the Shannon entropy. IEEE Trans. Inf. Theory.

[39] Cover T., Thomas J. (2006). Elements of Information Theory.

[40] Endres D., Schindelin J. (2003). A new metric for probability distributions. IEEE Trans. Inf. Theory.

[41] Calkins H., Reynolds M.R., Spector P., Sondhi M., Xu Y., Martin A., Williams C.J., Sledge I. (2009). Treatment of atrial fibrillation with antiarrhythmic drugs or radiofrequency ablation: Two systematic literature reviews and meta-analyses. Circ. Arrhythm. Electrophysiol..

[42] Sotomi Y., Inoue K., Ito N., Kimura R., Toyoshima Y., Masuda M., Iwakura K., Fujii K. (2013). Incidence and risk factors for very late recurrence of atrial fibrillation after radiofrequency catheter ablation. Europace.

[43] Andrade J.G., Khairy P., Macle L., Packer D.L., Lehmann J.W., Holcomb R.G., Ruskin J.N., Dubuc M. (2014). Incidence and significance of early recurrences of atrial fibrillation after cryoballoon ablation: Insights from the multicenter Sustained Treatment of Paroxysmal Atrial Fibrillation (STOP AF) Trial. Circ. Arrhythm. Electrophysiol..

[44] Arora R., Verheule S., Scott L., Navarrete A., Katari V., Wilson E., Vaz D., Olgin J.E. (2003). Arrhythmogenic substrate of the pulmonary veins assessed by high-resolution optical mapping. Circulation.

[45] Hsieh M.H., Tai C.T., Lee S.H., Lin Y.K., Tsao H.M., Chang S.L., Lin Y.J., Wongchaoen W., Lee K.T., Chen S.A. (2006). The different mechanisms between late and very late recurrences of atrial fibrillation in patients undergoing a repeated catheter ablation. J. Cardiovasc. Electrophysiol..

[46] Berenfeld O., Zaitsev A.V., Mironov S.F., Pertsov A.M., Jalife J. (2002). Frequency-dependent breakdown of wave propagation into fibrillatory conduction across the pectinate muscle network in the isolated sheep right atrium. Circ. Res..

[47] Cervigón R., Moreno J., García-Quintanilla J., Pérez-Villacastín J., Castells F. (2016). Entropy at the right atrium as a predictor of atrial fibrillation recurrence outcome after pulmonary vein ablation. Biomed. Technol..

[48] Ho S.Y., Sánchez-Quintana D., Becker A.E. (2004). A review of the coronary venous system: A road less travelled. Heart Rhythm.

[49] Karagueuzian H.S. (2010). Triggered activity, coronary sinus, and atrial fibrillation. Heart Rhythm.

